# Methotrexate in Atherogenesis and Cholesterol Metabolism

**DOI:** 10.1155/2011/503028

**Published:** 2011-02-22

**Authors:** Eric Coomes, Edwin S. L. Chan, Allison B. Reiss

**Affiliations:** ^1^Department of Medicine, Division of Translational Medicine, New York University School of Medicine, NBV 16N1, 550 First Avenue, New York, NY 10016, USA; ^2^Inflammation Section, Winthrop Research Institute, Department of Medicine, Winthrop University Hospital, 222 Station Plaza North, Suite 502, Mineola, NY 11501-3893, USA

## Abstract

Methotrexate is a disease-modifying antirheumatic drug commonly used to treat inflammatory conditions such as rheumatoid arthritis which itself is linked to increased cardiovascular risk. Treatments that target inflammation may also impact the cardiovascular system. While methotrexate improves cardiovascular risk, inhibition of the cyclooxygenase (COX)-2 enzyme promotes atherosclerosis. These opposing cardiovascular influences may arise from differing effects on the expression of proteins involved in cholesterol homeostasis. These proteins, ATP-binding cassette transporter (ABC) A1 and cholesterol 27-hydroxylase, facilitate cellular cholesterol efflux and defend against cholesterol overload. Methotrexate upregulates expression of cholesterol 27-hydroxylase and ABCA1 via adenosine release, while COX-2 inhibition downregulates these proteins. Adenosine, acting through the A_2A_ and A_3_ receptors, may upregulate proteins involved in reverse cholesterol transport by cAMP-PKA-CREB activation and STAT inhibition, respectively. Elucidating underlying cardiovascular mechanisms of these drugs provides a framework for developing novel cardioprotective anti-inflammatory medications, such as selective A_2A_ receptor agonists.

## 1. Introduction

Rheumatoid arthritis is a multisystem autoimmune disorder [1]. Inflammatory processes in rheumatoid arthritis lead to accelerated atherosclerosis and afflicted patients have an elevated risk for cardiovascular events leading to a higher mortality [1]. In particular, elevated levels of immune complexes and cytokines (tumor necrosis factors, interleukins, and interferons (IFN)) contribute to the pathogenesis of both the articular [2] and cardiovascular manifestations of rheumatoid arthritis [3]. As such, the cardiovascular actions of antiarthritic medications are an important determinant for multiple aspects of patient care [3]. 

Although traditional nonsteroidal anti-inflammatory drugs (NSAIDs) [4], cyclooxygenase (COX)-2 inhibitors [5], methotrexate [6], and other disease-modifying antirheumatic drugs (DMARDs) are effective treatments for pain and inflammation, each of these drugs is linked with particular adverse effects which necessitate consideration. While NSAIDs are associated with gastrointestinal complications [7], some COX-2 inhibitors are associated with an elevated risk of cardiovascular events [5]. The COX-2 inhibitor cardiovascular risk has resulted in a market recall of several of these medications [8]. Although still associated with some adverse effects, methotrexate has proven to be one of the safest antirheumatic drugs [9].

In contrast to COX-2 inhibitors, methotrexate has demonstrated atheroprotective properties [10]. Recent studies have begun unravelling the molecular interactions underpinning the cardiovascular influence of COX-2 inhibition [11, 12] and methotrexate [13]. While these drugs have multiple cellular effects, cardiovascular modulation in part relies upon the regulation of reverse cholesterol transport [13, 14]. This review aims to provide an overview of the mechanisms by which methotrexate interacts with cholesterol homeostasis to modulate atherogenesis in inflammatory disease.

## 2. Mechanism of Action of Methotrexate

Methotrexate is an anti-inflammatory medication commonly used in the treatment of rheumatoid arthritis and other inflammatory diseases such as psoriasis and inflammatory bowel disease [9]. The primary anti-inflammatory actions of methotrexate are attributable to adenosine release, triggered by methotrexate's polyglutamate metabolites [6]. 

Adenosine, a nucleoside produced by many cells and tissues in response to physical or metabolic stresses, is an endogenous anti-inflammatory mediator [15]. The physiological effects of adenosine are mediated by G-protein coupled 7-transmembrane receptors [16] that exist on almost all human cell types [15]. Adenosine receptors are divided into four classes, A_1_, A_2A_, A_2B_, and A_3_, based on the differential selectivity of adenosine analogues and molecular structure [15].

It has been noted that some of the anti-inflammatory effects of methotrexate may be reversed by inhibition of the A_2A_ receptor [17]. Further research suggests that stimulation of the A_2A_ receptor interferes with inflammatory processes such as the biosynthesis and release of proinflammatory cytokines, inhibits oxidative activity, prevents platelet aggregation, and reduces adhesion and degranulation of neutrophils [18]. Together these results demonstrate that the A_2A_ receptor plays a significant role in the mediation of inflammation [17, 18]. 

Although adenosine has anti-inflammatory properties principally through its A_2A _[17] and A_3_ [19] receptors, it can also act in contradictory fashions via alternate receptors [20]. Ligation of adenosine to the A_1_ receptor initiates inflammatory processes [20]. The adenosine receptors influence inflammatory processes by modulating cAMP signalling cascades [20]. In particular, the opposing effects of A_2A_ and A_1 _activation are accounted for, respectively, by an elevation or reduction in intracellular cAMP [20]. Consequently, the overall effect of adenosine on inflammatory processes depends upon the relative temporal and spatial distribution of the various adenosine receptors, as well as the influence of other effectors in the inflammatory milieu [20].

## 3. Atherosclerosis and Reverse Cholesterol Transport

### 3.1. Atherosclerotic Development

Atherosclerosis, an underlying cause of myocardial infarction and stroke, is an intricate process that comprises elements of both inflammation and lipid accumulation, characterized by the thickening of arterial walls due to the development of a fibrous plaque [21]. When lipid load processing is inadequate in the monocyte infiltrated subendothelial intimia [22], unregulated cholesterol depositions in macrophages transform them into foam cells, marking the formation of fatty streaks [21]. Subsequent development forms a fibrous plaque narrowing the arterial lumen [21].

### 3.2. Reverse Cholesterol Transport

One of the initiating factors in the development of atherosclerosis is a deregulation of cholesterol homeostatic mechanisms [23]. The metabolism and removal of excess cholesterol is required to prevent foam cell accumulation [24]. Reverse cholesterol transport (RCT) is the process that transports such cholesterol from extrahepatic cells to the liver and intestine for excretion [25]. By preventing lipid accumulation via the control of cholesterol efflux, RCT pathways provide a defensive mechanism against proatherogenic cholesterol overload [25]. 

Extrahepatic cells eliminate intracellular cholesterol via multiple mechanisms of RCT, including adenosine triphosphate binding cassette transporter (ABC) A1 [26], ABCG1 [27], and cholesterol 27-hydroxylase (27-hydroxylase) [28] pathways. Cholesterol may be removed to HDL using ABCA1 [26] and ABCG1 [27] or processed within the cell by 27-hydroxylase prior to passive diffusion [28].

### 3.3. ABCA1: Adenosine Triphosphate-Binding Cassette Transporter A1

Excess cholesterol within the cell may be actively transported outward to maintain cholesterol balance [25]. In particular, ABCA1 acts as a rate-controlling transporter in this cholesterol efflux process [26]. Interactions of apolipoproteins with cholesterol-loaded cells stimulate the ABCA1 mediated transport of cholesterol and phospholipids to extracellular apoA-I [26]. A series of subsequent modifications ultimately lead to hepatic uptake and biliary excretion of cholesterol [29]. 

Mutations in ABCA1 cause a severe HDL deficiency syndrome known as Tangier disease [30]. The reduction in apoA-I initiated efflux of cholesterol leads to the accumulation of lipid deposits in tissues throughout the body [30]. Consequently, patients with this disease have a marked increase in the risk of coronary artery disease [31]. 

Although ABCA1 is normally constitutively expressed, in grossly atherosclerotic tissue, ABCA1 protein is reduced despite upregulation in ABCA1 mRNA [32]. This suggests that in the progression of atherosclerosis, posttranscriptional processes prevent the formation of ABCA1 [32]. Lee-Rueckert et al. [33] have demonstrated that ABCA1 expression is significantly attenuated by acidification. Since atherosclerotic tissue is gradually acidified as the lesion advances, this acid-dependent downregulation may partially account for the observed decrease in ABCA1 [34]. Given the association between ABCA1 mutation and atherosclerosis [31] as well as the observance of ABCA1 downregulation in atherogenesis [32], support is given to the notion that the dysregulation of ABCA1 facilitates the development of atherosclerosis.

### 3.4. Cholesterol 27-Hydroxylase

Although ABC-dependent RCT is the primary mechanism of cholesterol removal [26], the 27-hydroxylase pathway provides an alternative to the apoA-I-dependent process [28]. The mitochondrial cytochrome P450 27-hydroxylase enzyme is involved in the first oxidation step of cholesterol in the “acidic” pathway for biosynthesis of bile acids [35]. 27-hydroxylase is expressed at high levels in the THP-1 human monocytoid cell line, macrophages, and principal cell types in the atherogenic process such as human arterial endothelium and monocytes [35]. 

27-hydroxylase catalyzes the conversion of cholesterol to 27-hydroxycholesterol and subsequently 3-*β*-hydroxy-5-cholestenoic acid [36]. Effectively, this enzyme converts cholesterol into an oxygenated derivative or oxysterol. Such oxysterols are generally present in trace quantities, but their levels rise within atheromas [37]. The rapid excretion and degradation of these oxysterols is facilitated by their ability to pass lipophilic membranes [12, 38]. 

In atheroma, the dominant oxysterol is 27-hydroxycholesterol, at levels up to one-hundred times greater than in circulation [37, 39]. Given the faltering ability of ABCA1 to initiate RCT in advanced atheromas [32], the 27-hydroxylase mediated efflux pathway represents a major remaining defensive mechanism against excess cholesterol loading [38].

### 3.5. ABCA1 and 27-Hydroxylase Expression

At the level of gene expression, an interconnecting factor exists between ABCA1 and 27-hydroxylase [40, 41]. Oxysterols, including 27-hydroxycholesterol, bind and activate the liver X receptors (LXR) [40]. This interaction induces the nuclear translocation of an LXR-retinoid X receptor heterodimer, a transcription factor, which promotes the expression [42] of cholesterol metabolism genes, particularly ABCA1 [41]. 

## 4. Immunological and Inflammatory Processes in Atherogenesis

Accumulating evidence indicates that the systemic inflammatory load in lupus disrupts cholesterol dynamics, increasing vulnerability to cholesterol accumulation in cells of the artery wall, including macrophages and endothelium [43]. Patients with lupus [44] and rheumatoid arthritis [1] are known to have an increased risk for atheromatous cardiovascular disease. The immune-complex dysregulation seen in these conditions may play a role in the atherogenic process, a notion supported by evidence from cholesterol loading and efflux [44, 45]. 

In some inflammatory disease, antibodies to oxidized LDL are generated [45]. The resulting formation of oxLDL-antibody complexes enhances uptake of oxLDL into macrophages and promotes foam cell formation [44, 45]. Furthermore, stimulation of cultured human monocytes and THP-1 human monocytoid cells with the immune reactants IFN-*γ* or immune complexes impede cellular cholesterol efflux by markedly decreasing 27-hydroxylase and ABCA1 [46]. Collectively, these immune and inflammatory mediators promote atherosclerosis by disabling mechanisms that prevent the cells of the artery wall from being overloaded with cholesterol, leading to the formation of lipid laden foam cells [46, 47].

Research suggests that IFN-*γ* inhibits RCT by modifying signal transducer and activator of transcription (STAT) protein activity via two alternate mechanisms [48, 49]. In the first process, IFN-*γ* binds to the IFN receptor, initiating the dimerization of the two IFN-*γ* receptor sub-units which phosphorylates the bound janus kinases [48, 50]. The activated kinase phosphorylates the IFN-*γ* receptor, recruiting STAT proteins [50]. These STAT proteins are tyrosine phosphorylated, dimerize and translocate to the nucleus, where they downregulate LXR*α*, which in turn decreases ABCA1 expression [48]. 

In the second process, IFN-*γ* induces a calcium flux which activates the calcium/calmodulin-dependent protein kinase II (CaMKII) [49]. CaMKII directs the phosphorylation of the STAT residue, Ser^727^ which maximizes STAT activity [51]. The ability of both immune complexes and IFN-*γ* to influence RCT and macrophage to foam cell conversion emphasizes the importance of cardiovascular considerations in the treatment of inflammatory autoimmune disorders [47].

## 5. Mechanisms for Methotrexate Influence on Reverse Cholesterol Transport and Atherosclerosis

Across a multiplicity of reviewed studies, COX-2 inhibition elevated cardiovascular risk [5] while methotrexate significantly reduces cardiovascular disease mortality amongst rheumatoid arthritis patients [10], despite some disagreement for patients with prior atherosclerotic development [52]. Given the fundamental role of ABCA1 and 27-hydroxylase in regulating foam cell formation, we postulated a potential role for RCT in the atherosclerotic effects following treatment with COX-2 inhibitors and methotrexate [13]. The application of COX-2 inhibitors to THP-1 monocytes cultures resulted in significant decreases in the gene expression of 27-hydroxylase and ABCA1 [14]. The resultant reduction in ABCA1 and 27-hydroxylase protein creates an environment where cholesterol efflux is compromised, promoting foam cell formation [14]. 

Significantly, the effects of COX-2 inhibition on cholesterol metabolism and efflux may be reversed by the addition of adenosine A_2A_ receptor agonists [11]. Given the previously described ability of methotrexate to induce adenosine release [6], it is plausible that methotrexate is capable of upregulating RCT as its mechanism for atheroprotection. 

Using RT-PCR and immunoblotting, the levels of expression of ABCA1 and 27-hydroxylase were evaluated in cells in the presence of COX-2 inhibitors or IFN-*γ* with or without methotrexate [13]. Methotrexate reversed foam cell formation and the down regulation of ABCA1 and 27-hydroxylase induced by COX-2 inhibition or IFN-*γ* [13]. However, this methotrexate-induced upregulation was prevented by an inhibition of adenosine A_2A_ receptors [13]. Thus, the capacity of methotrexate to reduce the onset of atherosclerosis may be partially attributed to the adenosine-driven upregulation of ABCA1 and 27-hydroxylase which expedites cholesterol efflux [13].

**Figure 1:**
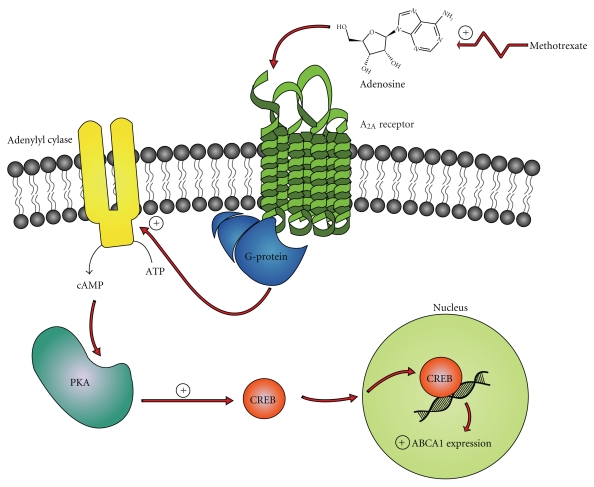
Regulation of adenosine triphosphate binding cassette transporter A1 (ABCA1) expression by adenosine. Methotrexate via numerous steps (jagged arrow) results in increased levels of adenosine. Adenosine activates the G-protein coupled receptor, A_2A_, inducing an increase in adenylyl cyclase activity. The subsequent rise in cyclic adenosine monophosphate (cAMP) activates protein kinase A (PKA) which phosphorylates the cAMP response element-binding protein (CREB). CREB then translocates into the nucleus where it upregulates ABCA1 gene expression.

The specific mechanism by which adenosine A_2A_ receptor ligation modifies 27-hydroxylase and ABCA1 expression has not yet been elucidated [12]. However, the inhibition of PKA prevents adenosine A_2A_ stimulation of RCT proteins, demonstrating a dependency upon a cAMP-PKA-mediated process [46]. Generally, A_2A_ receptor ligation activates adenylate cyclase, leading to the accumulation of cAMP [53]. cAMP activates PKA which subsequently activates CREB proteins [46]. These proteins then translocate into the nucleus, where it interacts with a consensus element in the gene promoter, modulating the expression of the target gene [46] (Figure 1).

As previously discussed, 27-hydroxycholesterol upregulates ABCA1 expression [40, 41]. A reasonable hypothesis is that ABCA1 upregulation is dependent upon the increased expression of 27-hydoxylase. However, inhibition of the 27-hydroxylase enzyme does not hinder the ability of adenosine A_2A_ ligation to upregulate ABCA1, suggesting that the methotrexate-induced increase in ABCA1 expression is not contingent on this feedback loop [46].

In addition to stimulating increased ABCA1 expression, A_2A_ ligation augments ABCA1 activity [54]. The generation of cAMP by A_2A_ stimulation activates protein kinases and Epac [54], a guanine nucleotide exchange factor [55]. Protein kinase generates phospho-ABCA1 and Epac activates ABCA1 function [54]. Thus, methotrexate promotes cholesterol efflux and hinders atherogenesis by both upregulating and activating RCT proteins. 

**Figure 2:**
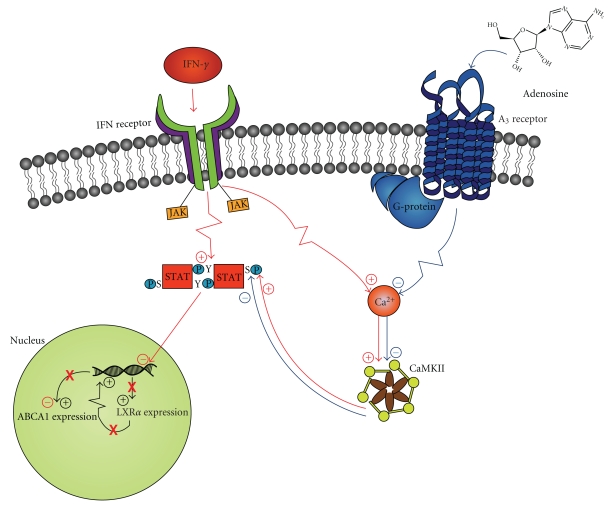
Abrogation of interferon (IFN)-*γ* mediated downregulation of adenosine triphosphate binding cassette transporter A1 (ABCA1) by adenosine A_3_ receptor ligation. IFN-*γ* binds the IFN receptor and subsequently decreases ABCA1 expression by increasing signal transducer and activator of transcription (STAT) protein activity via tyrosine (Y) and serine (S) phosphorylation. The IFN receptor induces a janus-kinase mediated process which tyrosine phosphorylates STAT. The serine is phosphorylated by a calcium/calmodulin-dependent protein kinase II (CaMKII) activated by a calcium flux initiated by the IFN receptor. These activated STAT proteins dimerize and translocate to the nucleus, where they downregulate liver X receptor (LXR)*α*. While LXR normally upregulates ABCA1 expression, a decrease in LXR results in a consequent decrease in ABCA1 expression. Significantly, adenosine may counteract the effects of IFN-*γ* on ABCA1 via the A_3_ receptor. A_3_ activation prevents the accumulation of Ca^2+^ and may thus prevent the activation of CaMKII. This reduced activity will decrease the phosphorylation of S^727^ on STAT, decreasing STAT activity and thus preventing the downregulation of ABCA1.

Beyond the A_2A_ receptor, adenosine may counteract the effects of IFN-*γ* via the A_3_ receptor [49]. The addition of adenosine to IFN-*γ* stimulated cells decreases ser^727^ phosphorylation, thereby reducing STAT activity [49]. This decrease in STAT activity decreases the expression of genes involved in inflammation and lipid uptake [48]. Inhibition of the A_3_ receptor reverses the suppressive effects of adenosine on STAT ser^727^ phosphorylation [49]. Given the role of STAT in suppressing RCT [48], this activity may additionally contribute to the antiatherosclerotic actions of methotrexate [49]. Although the A_3_-mediated pathway has yet to be uncovered, it has been demonstrated that A_3_ receptor activation prevents the accumulation of Ca^2+^ [56]. Thus, it is possible that A_3_ activity prevents the activation of CaMKII, which subsequently reduces the phosphorylation of ser^727^ [49] (Figure 2).

## 6. Conclusion

While methotrexate improves cardiovascular risk in a number of inflammatory diseases [10], COX-2 inhibition promotes atherosclerosis [5]. These opposing cardiovascular influences of methotrexate and COX-2 inhibitors may arise from differing effects on the expression of ABCA1 and 27-hydroxylase, key proteins in RCT [25]. While methotrexate upregulates their expression [13], COX-2 inhibition causes a downregulation [14]. Methotrexate increases the expression of these proteins via adenosine acting upon the A_2A_ [13] and A_3_ receptors [49]. Activation of A_2A_ increases expression of the proteins and activity of ABCA1 by a cAMP-mediated process [46] while the A_3_ receptor counteracts the IFN-*γ* induced downregulation by inhibiting STAT [49]. Furthermore, as proposed in the Cardiovascular Inflammation Reduction Trial, methotrexate administration may provide additional antiatherosclerotic benefits in patients already receiving traditional cholesterol-lowering therapy, regardless of inflammatory disease status [57]. Thus, the understanding of the mechanisms by which these medications act to induce cardiovascular effects provides a platform for the design of novel and safer anti-inflammatory and antiatherosclerotic drugs in the future, such as selective adenosine A_2A_ receptor agonists.

##  Declaration

E.S.L. Chan declares that he holds a patent pertinent to King Pharmaceuticals on the use of adenosine A_2A_ receptor antagonists to inhibit fibrosis.

## Abbreviations

IFN: Interferon
NSAID: Nonsteroidal anti-inflammatory drug
COX: Cyclooxygenase
DMARD: Disease modifying antirheumatic drug
cAMP: Cyclic adenosine monophosphate
RCT: Reverse cholesterol transport
ABC: Adenosine triphosphate binding cassette transporter
HDL: High density lipoprotein
apoA-I: Apolipoprotein AI
mRNA: Messenger ribonucleic acid
THP-1: Human acute monocytic leukemia cell line
LXR: Liver X receptor
ox-LDL: Oxidized low density lipoprotein
STAT: Signal transducer and activator of transcription
PKA: Calcium/calmodulin-dependent protein kinase II, protein kinase A
CREB: cAMP response element-binding
ATP: Adenosine triphosphate
Epac: Exchange protein activated by cAMP.
